# 4mCpred-EL: An Ensemble Learning Framework for Identification of DNA *N^4^*-Methylcytosine Sites in the Mouse Genome

**DOI:** 10.3390/cells8111332

**Published:** 2019-10-28

**Authors:** Balachandran Manavalan, Shaherin Basith, Tae Hwan Shin, Da Yeon Lee, Leyi Wei, Gwang Lee

**Affiliations:** 1 Department of Physiology, Ajou University School of Medicine, Suwon 16499, Korea; bala@ajou.ac.kr (B.M.); shaherinb@gmail.com (S.B.); catholicon@ajou.ac.kr (T.H.S.); ekdus93@ajou.ac.kr (D.Y.L.); 2 School of Software, Shandong University, Jinan 250101, China

**Keywords:** machine learning, DNA methylation, mouse genome, *N^4^*-methylcytosine identification

## Abstract

DNA *N*^4^-methylcytosine (4mC) is one of the key epigenetic alterations, playing essential roles in DNA replication, differentiation, cell cycle, and gene expression. To better understand 4mC biological functions, it is crucial to gain knowledge on its genomic distribution. In recent times, few computational studies, in particular machine learning (ML) approaches have been applied in the prediction of 4mC site predictions. Although ML-based methods are promising for 4mC identification in other species, none are available for detecting 4mCs in the mouse genome. Our novel computational approach, called 4mCpred-EL, is the first method for identifying 4mC sites in the mouse genome where four different ML algorithms with a wide range of seven feature encodings are utilized. Subsequently, those feature encodings predicted probabilistic values are used as a feature vector and are once again inputted to ML algorithms, whose corresponding models are integrated into ensemble learning. Our benchmarking results demonstrated that 4mCpred-EL achieved an accuracy and MCC values of 0.795 and 0.591, which significantly outperformed seven other classifiers by more than 1.5–5.9% and 3.2–11.7%, respectively. Additionally, 4mCpred-EL attained an overall accuracy of 79.80%, which is 1.8–5.1% higher than that yielded by seven other classifiers in the independent evaluation. We provided a user-friendly web server, namely 4mCpred-EL which could be implemented as a pre-screening tool for the identification of potential 4mC sites in the mouse genome.

## 1. Introduction

Dynamic DNA modifications, such as methylation and demethylation play crucial roles in the regulation of gene expression. Methylation of cytosine at *CpG* sites is considered as an important epigenetic mark that is involved in the regulation of cell differentiation, genomic imprinting, cell cycle, aging, preservation of chromosome stability, and gene expression levels [1,2]. The three common cytosine methylations identified in both prokaryotic and eukaryotic genomes are *N*^4^-methylcytosine (4mC), 5-methylcytosine (5mC) (mediated enzymatically via DNA methyltransferases), and 3-methylcytosine, (produced upon exposure to endogenous or environmental alkylation agents) [3,4]. Due to the widespread distribution and multi-faceted roles of 5mC, it is the most well-explored and common type of cytosine methylation that illustrates a significant role in several biological processes [5,6] and is associated with neurological diseases, diabetes, and cancer [7,8,9]. 4mC is also regarded as a potent epigenetic modification that protects its self-DNA from the restriction enzyme-mediated degradation. However, experimental studies on 4mC have lagged owing to the paucity of effective identification approaches. Furthermore, the exact mechanisms of epigenetic modifications and biological functions of 4mC sites are also limited.

Although 4mC is less investigated compared to 5mC, it has varied tasks in controlling DNA replication, differentiating self and non-self-DNA, cell cycle, correcting DNA replication errors, and gene expression levels [10,11]. To identify epigenetic cytosine nucleobases, several experimental approaches, such as methylation-specific PCR, mass spectrometry, whole-genome bisulphite sequencing, the engineered transcription-activator-like effectors (TALEs) approach, reduced-representation bisulphite sequencing, and single-molecule real-time sequencing (SMRT) are available [12,13,14]. Although these experimental techniques assist in the detection of cytosine methylation sites, they are expensive and time-consuming when applied on large-scale genome sequencing. Therefore, a pressing need exists for the development of efficient computational methods for 4mC sites identification.

Lately, several 4mC sites prediction methods have been proposed for six different species, namely *Geoalkalibacter subterraneus*, *Arabidopsis thaliana*, *Geobacter pickeringii*, *Escherichia coli*, *Drosophila melanogaster*, and *Caenorhabditis elegans* [15,16,17,18,19]. The state-of-the-art ML approaches for 4mC site predictions demonstrated the necessity of species-specific models employing cross-species evaluation. All of these approaches utilized positive samples (4mC sites) obtained from the MethSMRT database [20], which was developed by integrating the publicly available SMRT sequencing datasets. Unfortunately, there is no 4mC sites prediction method available for the mammalian mouse genome. In general, mouse serves as a well-established experimental animal model, since it has been used to mimic the effect of epigenetic modifications related to mammalian development and human diseases [21,22], and it has virtually the same set of genes as humans. Therefore, it is necessitated to develop a novel ML-based predictor for the detection of 4mC sites in the mouse genome.

Here, a new ensemble predictor, 4mCpred-EL has been established for the identification of 4mC sites in the mouse genome. Firstly, 28 probabilistic features were generated by employing four ML algorithms, namely gradient boosting (GB), extremely randomised tree (ERT), support vector machine (SVM) and random forest (RF), and seven feature encodings (binary profile (BPF), electron–ion interaction pseudopotentials (EIIP) of trinucleotides, a combination of dinucleotide binary encoding (DPE), and local position–specific dinucleotide frequency (LPDF) represented as M6AMRFS, k-mer composition (Kmer), ring-function-hydrogen-chemical properties (RFHC), dinucleotide-(DPCP) and trinucleotide-physicochemical properties (TPCP). Secondly, these probabilistic features were inputted to four different ML classifiers and their corresponding models were developed, which were then utilised for the generation of an ensemble predictor. Further validation of 4mCpred-EL was evaluated using an independent dataset. Our results demonstrated that the proposed model outperformed seven other ML-classifiers with higher prediction accuracies. Furthermore, we established a user-friendly online web server called 4mCpred-EL for the easy detection of 4mC sites from the mouse genome. We believe that this study represents the first 4mC site prediction method for the mouse genome, that will facilitate the identification of correct 4mC sites prediction and could be possibly extended to other species as well.

## 2. Materials and Methods

**Figure 1:**
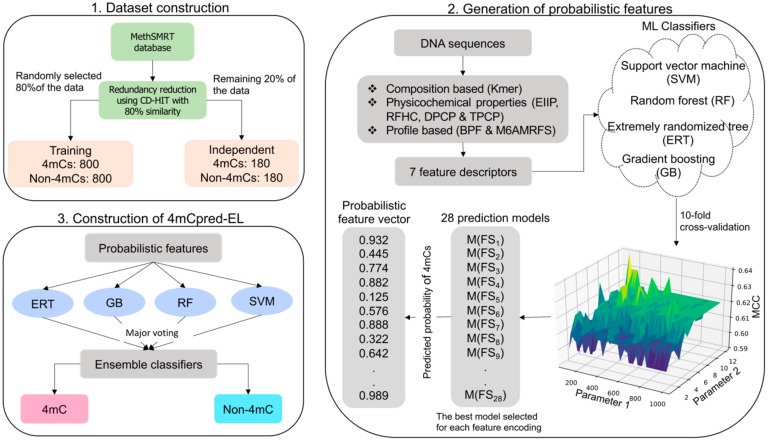
Schematic framework of 4mCpred-EL. It involves the following steps: (**1**) Dataset construction for the mouse genome, (**2**) Generation of probabilistic features using varied ML classifiers and seven feature encodings, and (**3**) Final model construction that discriminates the input into 4mC or non-4mC.

The overall framework of 4mCpred-EL was summarized as per Chou’s 5-step rule [23] (Figure 1): (i) benchmark and independent datasets were constructed; (ii) DNA sequences were mapped to the fixed length numerical vector, that could be further utilized as an input feature vector to the classifier; (iii) an effectual and appropriate classifier was employed to develop a model; (iv) cross-validations (CV) and evaluations were performed on the independent data to measure the reliability of the prediction model; and (v) an accessible web-server was established.

### 2.1. Dataset Construction

The positive samples (sequences comprising 4mC sites) of mouse genome were taken from the MethSMRT database [20]. All sequences contained a central cytosine (C) nucleotide (NT) with a length of 41 base pairs (bp). The following criteria were applied to generate a high-quality dataset: (i) only sequences that had a modQV score greater than 20 were considered, whereas the remaining sequences were excluded, which is in accordance with the Methylome Analysis Technical Note [24] (ii) to reduce homology bias, redundant sequences present in the collected samples were excluded via CD-HIT program [25], and by employing a sequence-identify cut-off of 0.8. Following the filtering, a non-redundant set of 980 positive samples was obtained. Notably, using a lower sequence identity threshold, such as 40% or 50%, might aid in reducing the bias and yield more reliable models. Nevertheless, the limited dataset size in the present study demanded the utility of CD-HIT with a higher cut-off.

The negative samples, i.e., sequences not containing 4mC sites, were obtained from chromosomal DNA sequences with a length of 41 bp and a C at their center that was not detected as 4mC by SMRT. Redundant sequences in the negative samples were subsequently removed by applying a CD-HIT cut-off value of 0.6. Negative samples that shared a sequence identity >60% with the positive samples were also removed. This procedure resulted in a large number of negative samples. Nonetheless, 980 samples were randomly selected, and a balanced dataset was generated. From the total pool of samples, ~80% samples were randomly selected (4mCs: 800 and non-4mCs: 800) and considered as the benchmark dataset for the prediction model development, whereas the rest of the samples (4mCs: 180 and non-4mCs: 180) were used as an independent set for the model validation.

### 2.2. Feature Extraction

Generally, an input sequence (DNA) is converted into a fixed length of feature vectors that could be handled by ML classifiers. Here, seven feature encodings, viz. BPF, DPCP, EIIP of trinucleotides, Kmer, M6AMRFS, RFHC, and TPCP, were used as the input for ML classifiers to discriminate 4mC-containing samples from those not containing 4mC sites. A succinct explanation of the feature extraction protocol is presented in the subsequent sections.

#### 2.2.1. Kmer

Kmer encoding is one of the simplest approaches for representing DNA sequences: it refers to the occurrence frequency of all the possible substrings of a length *k* that are contained in a DNA sequence, which has been applied for several prediction problems [26,27,28]. In a sequential model, a DNA sequence is expressed as: $=K_{1}K_{2}K_{3}\ldots K_{N}$, where *K_1_*, *K_2_*, and *K_3_* denote the first, second, third NT, and so on, and *N* indicates the sequence length. Notably, *K_i_* is one of the standard NTs, adenine (A), cytosine (C), guanine (G), or thymine (T). Here, mono-, di-, tri-, tetra-, and penta-NT compositions were considered and combined to form a 1364 ($4^{1}$ + $4^{2}$ + $4^{3}$ + $4^{4}$ + $4^{5}$) dimensional feature vector.

#### 2.2.2. M6AMRFS

It is a combination of DPE and LPDF, which has been successfully implemented in M6AMRFS method for N6-methyl adenosine prediction from RNA sequences [17,29]. In DPE, each 16 possible dinucleotide is encoded as 0/1 (four-dimensional vector). For instance, AT, AA, GG, and AC are respectively encoded as (0,0,0,1), (0,0,0,0), (1,1,1,1) and (0,0,1,0). Using DPE, a given sequence is converted to a 160(40bp × 4)-dimensional vector. LPDF descriptor is denoted as ($f_{2},f_{3},\;\ldots ,f_{j}$), where $f_{j}$ is computed as: $f_{j}=\frac{1}{\left|Y_{j}\right|}D\left(M_{j-1}M_{j}\right),\;2\leq j\leq N$, where |$Y_{j}$| is the length of the *j*th prefix string {*M_1_M_2_…M_j_*} in the sequence, and *D*($M_{j-1}M_{j}$) represents the frequency of the dinucleotide $M_{j-1}M_{j}$ in position *j* of the *j*th prefix string.

#### 2.2.3. RFHC

Based on the hydrogen bonds, functional groups, and ring structure, four nucleotides can be classified into three groups, which has been widely utilized for several problems [16,30,31]. Briefly, A and C containing the amino group, whereas G and T containing the keto group; two rings present in A and G, while C and T have only one ring; and C and G belong to one group due to formation of strong hydrogen bonds, while A and T are grouped as they form weak hydrogen bonds. To quantify these chemical properties, a given DNA sequence was represented with a 4-dimensional vector (*a*, *b*, *c*, *d_j_*), where *a*, *b*, and *c* are computed as follows:

(1) $$a=\left\{\begin{array}{l} 1\;\;\;\;\;\;\;if\;x\in \left\{A,G\right\}\\ 0\;\;\;\;\;\;\;\;\;\;\;\;\;\;\;\;\;others \end{array}\right.\;\;\;\;\;\;\;\;b=\left\{\begin{array}{l} 1\;\;\;\;\;\;if\;x\in \left\{A,T\right\}\\ 0\;\;\;\;\;\;\;\;\;\;\;\;\;\;\;\;others \end{array}\right.\;c=\left\{\begin{array}{l} 1\;\;\;\;if\;x\in \left\{A,C\right\}\\ 0\;\;\;\;\;\;\;\;\;\;\;\;\;\;others \end{array}\right.$$

Based on Equation (1), A, T, G, and C are symbolized as (1,1,1), (0,1,0) (1,0,0), and (0,0,1), respectively. Density $d_{i}$ of the NT in a DNA sequence is derived as:

(2) $$d_{j}=\frac{1}{\left|L_{j}\right|}\overset{N}{\underset{j=1}{\sum }}f\left(L_{j}\right),\;j=1,2,3,\cdots ,N\;;\;f\left(j\right)=\left\{\begin{array}{l} 1\;\;\;\;\;\;\;\;\;\;if\;L_{j}=p\\ 0\;\;\;\;\;\;\;\;\;\;\;\;\;\;\;\;\;\;\;else \end{array}\right.\;,\;p\in \left\{A,C,G,T\right\}$$

where $\left|L_{j}\right|$ is the length of the substring from the position *j* to the first position in the DNA sequence.

#### 2.2.4. EIIP of Trinucleotides

Nair and Sreedharan [32] proposed the distribution of electron–ion energies (EIIPs) along the DNA sequence that has been widely used in enhancer prediction [33], and nucleosome identification [34]. Namely, A, C, G, and T, respectively, have EIIP values of 0.1260, 0.1340, 0.0806, and 0.1335 [32]. The EIIP of trinucleotides encodes a 64-dimensional vector for a given DNA sequence as:

(3) $$V=\left[EIIP_{TTT}\cdot f_{TTT},EIIP_{GGG}\cdot f_{GGG},\cdots ,EIIP_{CCC}\cdot f_{CCC}\right]$$

The subscripts in Equation (3) correspond to various combinations of trinucleotides; $EIIP_{abc}=\;EIIP_{a}+EIIP_{b}+\;EIIP_{c}$, where $EIIP_{a}$ is the EIIP value of the corresponding nucleotide in the subscript, and *a*, *b*, *c* ∈ {A, G, C, T}; and $f_{abc}$ denotes trinucleotide (*abc*) normalized frequency.

#### 2.2.5. BPF

In BPF, the encoding of each NT is depicted as a feature vector of 0s and 1s. In particular, A is encoded as P(A) = (1, 0, 0, 0), T as P(T) = (0, 1, 0, 0), G as P(G) = (0, 0, 1, 0), and C as P(C) = (0, 0, 0, 1). For a given DNA sequence *D* with a fixed length of 41 NT, BPF could be encoded as:

(4) $$\mathrm{BPF}\left(L\right)=\left[P\left(K_{1}\right),P\left(K_{2}\right),P\left(K_{3}\right)\ldots P\left(K_{L}\right)\right]$$

Hence, the BFP(*L*) generates 164(4 × 41)-dimensional feature vector.

#### 2.2.6. DPCP

In this study, fifteen physicochemical properties were utilized in a manner like [18,27]. The Supplementary Table S1 presents an overview of these values for each dinucleotide normalized value. DPCP can be devised as:

(5) $$V=\left[DPCP_{AA}\cdot f_{AA},DPCP_{AC}\cdot f_{AC},\cdots ,DPCP_{TT}\cdot f_{TT}\right]$$

where *DPCP_i_* is one of the *i*th (*i* = 1,2,3…15) physicochemical properties of a dinucleotide listed in Supplementary Table S1, and $f_{\mathrm{NN}}$ denotes the normalized frequency of the dinucleotide. DPCP is translated as a 240-dimensional vector (from 16 dinucleotides × 15 physicochemical properties).

#### 2.2.7. TPCP

Eleven physicochemical properties were used similar to [18,27]. The normalized values of these properties for each trinucleotide is shown in Supplementary Table S2. TPCP can be devised as:

(6) $$V=\left[TPCP_{GGG}\cdot f_{GGG},TPCP_{GCA}\cdot f_{GCA},\cdots ,TPCP_{CCC}\cdot f_{CCC}\right]$$

where *TPCP_i_* is one of the *i*th (*i =* 1,2,3…11) physicochemical properties of a trinucleotide listed in Supplementary Table S2, and $f_{NNN}$ denotes the trinucleotide normalized frequency. TPCP is devised as a 704-dimensional vector (from 64 trinucleotides × 11 physicochemical properties).

### 2.3. Application of ML Algorithms in 4mCpred-EL

4mCpred-EL utilizes four commonly used ML classifiers, such as RF [35], GB [36], SVM [37], and ERT [38]. The Scikit-Learn package (v0.18) [39] was used to implement these classifiers in the current study. Notably, these classifiers have been widely utilized in the field of bioinformatics or computational biology [27,40,41,42,43,44,45,46,47,48,49,50,51,52,53,54,55,56]. Since the tuning parameters search ranges are different among four classifiers, we applied a grid search procedure and optimized each classifier separately. The computational procedure for prediction model development, including ML parameter search ranges for each classifier and 10-fold cross validation are similar to our recent studies [18,47,57,58,59,60].

### 2.4. Performance Evaluation

Specificity (SP), sensitivity (SN), Matthews correlation coefficient (MCC), and accuracy (ACC) were calculated to assess the performance of our proposed method [61].

(7) $$\left\{\begin{array}{l} SN=\frac{TP}{TP+FN}\\ SP=\frac{TN}{TN+FP}\\ ACC=\frac{TP+FN}{TP+TN+FN+FP}\\ MCC=\frac{TP\times TN-FP\times FN}{\sqrt{\left(TP+FN\right)\left(TP+FP\right)\left(TN+FP\right)\left(TN+FN\right)}} \end{array}\right.$$

where TP and TN represent the number of 4mCs correctly predicted and non-4mCs correctly predicted, respectively, FN is the number of not correctly predicted 4mC samples, and FP is the number of not correctly predicted non-4mCs.

## 3. Results and Discussion

### 3.1. Evaluation of Nucleotide Composition Preference

**Figure 2:**
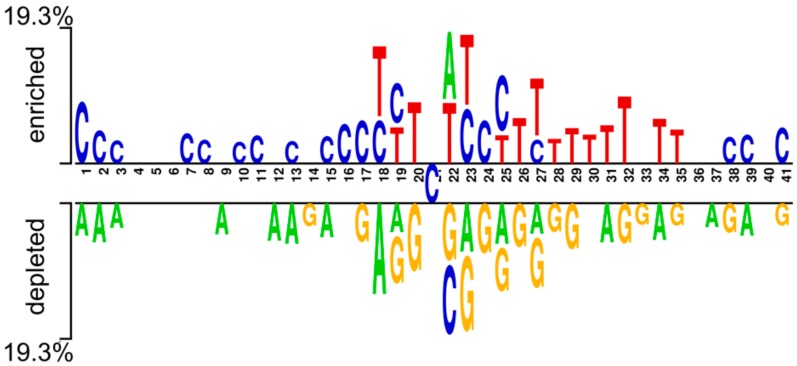
Compositional inclinations of sequences between 4mC and non-4mC sites. Logo mentioned above the axis indicates compositional preferences of positives, while the logo below the axis indicates compositional preferences of negatives.

To explore the DNA composition preferences between positives (4mC sites) and negatives (non-4mC sites), two-sample logos [62] were used to determine the statistically meaningful variations in position-specific composition. The following characteristics were observed (Figure 2): (i) both positives and negatives comprise the same C base in the middle of the sequences; (ii) C and T bases were respectively enriched at the upstream and downstream of the positives, while A and G were respectively depleted at the upstream and downstream of the negatives; (iii) some bases tended to appear successively along the sequences. For example, three consecutive C bases (positions 1 to 3 and 15 to 17) and four consecutive T bases (positions 28 to 32) were observed in positives, whereas three consecutive A bases (positions 1 to 3) were observed in negatives. Briefly, enriched and depleted nucleotides were considerably distinct between positives and negatives, demonstrating that bases around 4mC and non-4mC sites have position-specific preferences. Hence, it can be implied that the features with compositional and position data assists in the correct classification of true 4mC from non-4mC sites.

### 3.2. Performance Evaluation of Various ML Methods on Seven Feature Encodings

**Figure 3:**
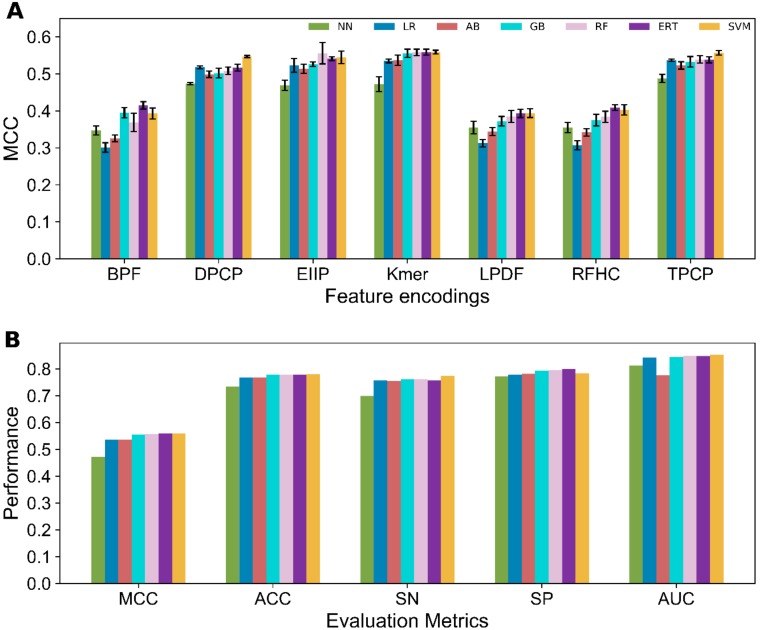
(**A**) Performances of seven classifiers with respect to seven feature encodings in terms of MCC. The best performance achieved by each classifier from seven encodings is shown in (**B**).

Seven different ML methods, namely GB, ERT, AdaBoosting (AB), *k*-nearest neighbor (KNN), logistic regression (LR), SVM, and RF were explored on various feature encodings to identify the ideal classifier for the 4mC site prediction problem. To test the generalizability of our approach, we carried out ten independent 10-fold CV test by randomly selecting 80% of the data from the original dataset and considered them as the benchmark dataset. Basically, each sample from the original data were utilized at least once during the prediction model development. Ten optimal models were developed with respect to each classifier and each encoding, whose evaluation metrices were averaged to depict their overall performance. The detailed performances of seven classifiers with respect to different feature encodings is shown in Supplementary Table S3 and briefly provided in Figure 3A. Results showed that the performance topology of 75% classifiers achieved MCC in the range 0.51–0.57 with respect to the four encodings (Kmer, EIIP of trinucleotides, DPCP, and TPCP), which is 15–16% higher than the remaining three encodings (M6AMRFS, BPF, and RFHC). Recently, only few methods implemented three encodings (M6AMRFS, BPF, and RF) in 4mC sites prediction for several prokaryotic and eukaryotic species. However, individually these features did not contribute much as we expected in the mammalian mouse genome [15,17], thus indicating that it can used as an additive. Interestingly, RF, GB, ERT, SVM, and AB achieved their best performance with an ACC in the range of 76.9–78.0% using Kmer. However, LR and KNN respectively achieved their best performances with an ACC of 76.8% and 73.6% using TPCP encodings (Figure 3B). Furthermore, the average performance of four classifiers (RF, ERT, GB and SVM) from seven encodings lies MCC in the range of 0.47–0.48 that consistently performed better than AB, LNN, and LR (MCC in the range of 0.42–0.44), thus signifying that decision tree-based and hyperplane-based methods are competitive with each other and better suited for this problem. Overall, none of the model achieved superior performance over other models, hence, we performed ensemble approach by considering only four classifiers (because of its consistency) and seven encodings.

### 3.3. Construction of 4mCpred-EL

**Figure 4:**
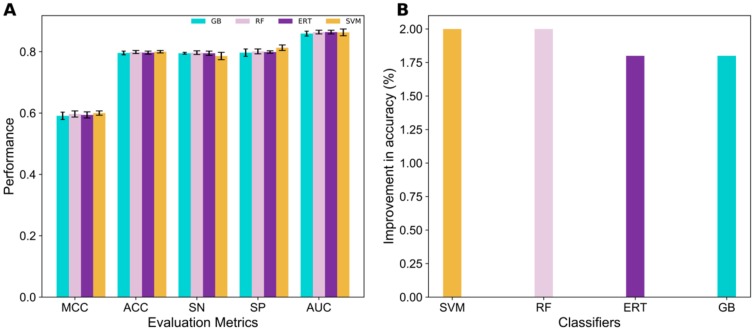
Meta-predictor performance and its improvement. (**A**) The performance comparison between four meta-predictors that utilize 28 probabilistic features as an input. (**B**) Improvement in the accuracy of each meta-predictor with respect to the baseline models.

Only four ML classifiers were considered, namely SVM, RF, ERT, and GB, due to their consistent performance on seven feature encodings, whose predicted probability of 4mC values lies in the range of 0.0–1.0. Here, if the predicted probability value was ≥ 0.50, it belonged to 4mCs, otherwise it belonged to non-4mCs. The predicted probability of 28 models (i.e., 7 feature encodings × 4 ML classifiers), which were considered as a feature vector, were inputted again to seven different classifiers to develop their corresponding prediction models or meta-predictors. Notably, each classifier contains 10 prediction models for individual encoding (as mentioned in the above section), hence, we utilized all these models and generated 10 meta-predictors for each classifier and the average performance is shown in Figure 4A. The results showed that RF, ERT, SVM, and GB achieved same performance, with accuracies in the range of 79.7–80.0%. Interestingly, each of the seven meta-predictors significantly improved the performance (Figure 4B) when compared to the best baseline models (Figure 3B), indicating the success of the meta-strategy combining the strength of multiple predictors and thereby improving the performances. This approach has been widely applied in various prediction problems previously [17,63].

Since the performance of ERT, GB, RF, and SVM were same, these predictors were further combined into an ensemble approach through a voting system;

E=ERT ∀ GB ∀ RF ∀ SVM

where E and ∀ respectively represented the ensemble classifier termed 4mCpred-EL and the fusing operator. 4mCpred-EL attained an MCC and ACC respectively of 0.591 and 79.5%. These metrics were similar when an individual meta-predictor was employed.

To verify the advantage of 4mCpred-EL, its performance was compared with the best models from the seven different classifiers, as shown in Figure 3B. Table 1 depicted that the MCC and ACC of 4mCpred-EL were 3.2–11.7% and 1.5–5.9% higher than those of the single models. Furthermore, the AUCs of 4mCpred-EL and other methods were compared, and the P-values were computed using a two-tailed *t*-test [64]. 4mCpred-EL considerably outshone most of the single models (except RF and SVM) with a P-value threshold of 0.05. Although several ML approaches have been quite commonly applied for 4mC site predictions in different species [15,65,66], this is the first ensemble approach developed for the identification of 4mCs in the mouse genome.

### 3.4. Feature Contribution and Relevance Analysis

**Figure 5:**
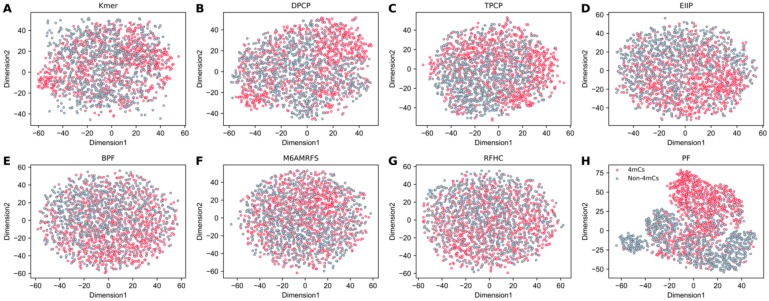
t-SNE visualization of the benchmark dataset in a two-dimensional feature space. Gray- and red-colored circles indicate non-4mCs and 4mCs, respectively. (**A**) Kmer, (**B**) dinucleotide (DPCP), (**C**) trinucleotide-physicochemical properties (TPCP), (**D**) EIIP of trinucleotides, (**E**) binary profile (BPF), (**F**) M6AMRFS, (**G**) ring-function-hydrogen-chemical properties (RFHC), and (**H**) the 28 probabilistic features (PF).

4mCpred-EL owes its improved performance mainly to the probabilistic features. To comprehend further, the *t*-distributed stochastic neighbor embedding (t-SNE) distributions of the positive (4mC) and negative (non-4mC) samples of the 28-dimensional probabilistic feature vector were computed and the results were compared using seven feature encodings, Kmer, RFHC, DPCP, TPCP, M6AMRFS, BPF, and EIIP of trinucleotides. The negative and positive samples distributions in the higher-dimensional space which can be represented by their mutual distances in a two-dimensional (2D) space is depicted in Figure 5. In Figure 5A–G, a clear inhomogeneity is observed in the distribution pattern of negative and positive samples, thus showing that the original feature discriminating power is less. On the contrary, an apparent distinction is observed for the probabilistic features (Figure 5H). This shows that negative and positive samples found in probabilistic features can be distinguished easily when compared to other features, thereby enriching the performance.

**Figure 6:**
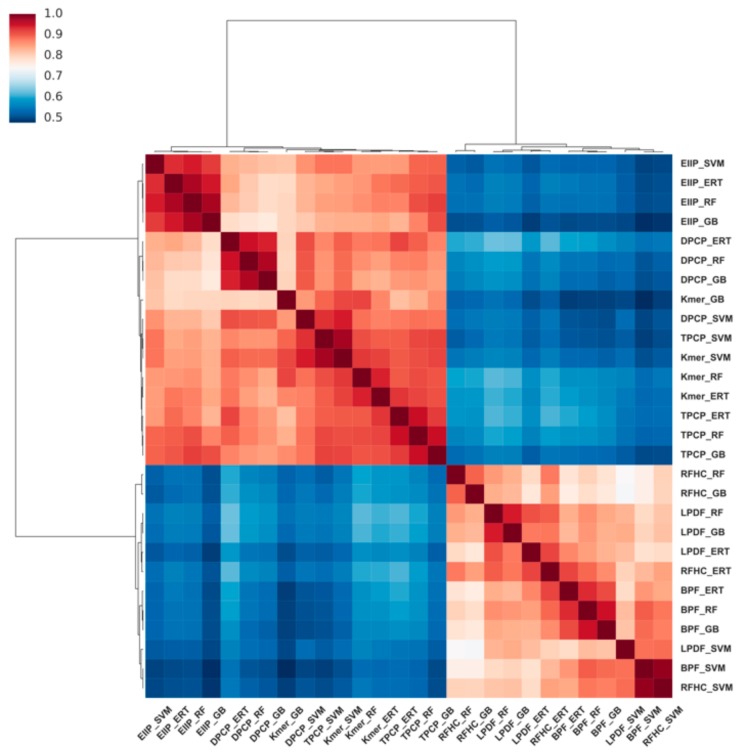
Cluster heatmap of the correlation between 28 probabilistic features. Dark red denotes very high correlation and dark blue denotes less correlation (correlation coefficient <0.50).

Next, the relationship between the 28 probabilistic features was investigated. To this end, the cluster heat maps of Pearson’s correlation coefficients between the 28 probabilistic features were plotted (Figure 6). The results demonstrated that the 28 probabilistic features were grouped into two clusters, where the first cluster contained 16 features (4 classifiers × 4 encodings (TPCP, EIIP of trinucleotides, Kmer, and DPCP)) that are highly correlated with each other, and the second cluster contained the remaining 12 features (4 classifiers × 3 encodings (M6AMRFS, BPF, and RFHC)), thereby indicating that these features are complementary in contributing to the predictive performance.

### 3.5. Evaluation of Various Methods on the Independent Dataset

It should be noted that 80% were randomly selected from the original data (980 4mCs and 980 non-4mCs) and considered as benchmark dataset and the remaining 20% were considered as independent dataset. Since, we repeated this process 10 times, 10 benchmark and 10 independent datasets were obtained. As mentioned above, basically 10 different benchmark datasets were employed and developed their corresponding prediction model for each approach as listed in Table 1. Therefore, all the 10 models were evaluated on the respective independent datasets and the average performance is shown in Table 2. Since this is the first such method, its performance was compared with only seven different baseline predictors (Figure 3) that produced the best performance among the seven different feature encodings.

Table 2 shows that 4mCpred-EL displayed the best performance with respect to MCC, ACC, and AUC (0.596, 79.8%, and 0.897, respectively). In particular, MCC and ACC of 4mCpred-EL were approximately 3.4–8.8% and 1.8–5.1% higher than those of individual classifiers, thus showing supremacy of the proposed method. Surprisingly, the benchmark and independent results listed in Table 1 and Table 2 is similar for each method, indicating the robustness of each classifier. Among them, 4mCpred-EL achieved a superior performance on both datasets, which is primarily due to the following aspects: (i) the probabilistic features integrate wide range of sequence information (including NT composition, ring function, NT position-specific information, and physicochemical properties) that distinguish positive and negative samples efficiently; (ii) integration of different classifiers by means of an ensemble approach by considering each classifier’s advantage and disadvantage to make a robust prediction model.

### 3.6. Web Server Implementation

For the convenience of researchers, we established a user-friendly web server that it is available at http:/thegleelab.org/4mCpred-EL. Particularly, 4mCpred-EL server implemented only the fifth model among 10 models developed in this study, which achieved consistent performance on both benchmark (MCC: 0.589 and ACC: 0.794) and independent datasets (MCC: 0.612 and ACC: 0.806) using our webserver. In order to retrieve the predicted results, a user has to adhere to the following steps: (i) Prior to the submission, query sequences should be inputted to the input box in FASTA format, for which example could be found by clicking on the FASTA format button above the right corner of the input box; (ii) Subsequently, the server outputs predicted results by clicking on the ‘Submit’ button.

## 4. Conclusions

In this study, the predictor, called 4mCpred-EL, has been proposed for identifying 4mC sites in the mouse genome. For the development of an efficient predictive model, 28 probabilistic features were initially generated using four individual classifiers and seven feature encodings that cover various aspects of sequence data, such as NT position-specific, compositional, physicochemical, and ring-function information. Consequently, these features were again inputted to ERT, SVM, RF, and GB and their corresponding meta-predictors were developed. Finally, these four classifiers were combined into an ensemble approach through a voting system for the final prediction. The performance comparisons of 4mCpred-EL and the seven individual classifiers, RF, ERT, GB, SVM, AB, LR, and KNN, on both benchmark and independent datasets revealed that 4mCpred-EL achieved significantly better performance in identifying 4mCs. The improved performance of this method is due to the following: (i) the probabilistic feature vector captured the discriminative distribution characteristics between 4mCs and non-4mCs, and (ii) the appropriate classifiers were identified by exploring various classifiers, which were further utilized in the development of an ensemble predictor.

In addition to the prediction of 4mCs, this computational framework, including model training, meta-predictor construction, and ensemble strategy for the final prediction, can act as a valuable guide and stimulate computational biologists to develop new computational approaches. Furthermore, integrating other informative features such as pseudo-nucleotide composition and multivariate mutual information, along with the exploration of other ML algorithms such as deep learning and neural networks, might further improve its performance [67,68,69,70]. We anticipate that this methodology and the web server will accelerate the identification of putative 4mCs and considerably aid experimentalists in the elucidation of other functional mechanisms. The limitation of our method is that only sequence information was utilized for the prediction of 4mC sites owing to its less experimental investigation. However, such limitation could be addressed in future when the gene expression data for 4mCs become available. Besides sequence information, we will include such data in our future studies for the improved and accurate prediction of 4mC sites using ML methods.

## Figures and Tables

**Table 1:** Evaluation assessment between our proposed method and individual classifiers based on a benchmark set.

Method	MCC	ACC	SN	SP	AUC	*p*-Value
4mCpred-EL	0.591 ± 0.001	0.795 ± 0.001	0.804 ± 0.002	0.787 ± 0.002	0.874 ± 0.001	**—**
SVM	0.559 ± 0.005	0.780 ± 0.002	0.775 ± 0.004	0.784 ± 0.005	0.854 ± 0.003	0.127
ERT	0.559 ± 0.008	0.779 ± 0.004	0.758 ± 0.008	0.800 ± 0.004	0.848 ± 0.005	**0.049**
RF	0.558 ± 0.009	0.779 ± 0.004	0.762 ± 0.014	0.796 ± 0.008	0.849 ± 0.007	0.058
GB	0.556 ± 0.011	0.778 ± 0.006	0.762 ± 0.011	0.793 ± 0.002	0.845 ± 0.010	**0.029**
AB	0.537 ± 0.014	0.769 ± 0.007	0.755 ± 0.010	0.782 ± 0.006	0.777 ± 0.008	**<0.000001**
LR	0.537 ± 0.003	0.768 ± 0.002	0.758 ± 0.003	0.778 ± 0.001	0.842 ± 0.004	**0.016602**
KNN	0.474 ± 0.003	0.736 ± 0.002	0.692 ± 0.010	0.780 ± 0.006	0.815 ± 0.003	**0.000023**

Note: *p* < 0.05 shows a statistically significant difference between 4mCpred-EL and other method that is depicted in bold. Values are expressed as mean ± standard deviation.

**Table 2:** Evaluation assessment between our proposed ensemble method and individual classifiers based on an independent test set.

Method	MCC	ACC	SN	SP	AUC	*p*-Value
4mCpred-EL	0.596 ± 0.022	0.798 ± 0.011	0.804 ± 0.012	0.792 ± 0.028	0.897 ± 0.008	—
RF	0.562 ± 0.010	0.780 ± 0.005	0.736 ± 0.008	0.824 ± 0.014	0.862 ± 0.002	0.179
ERT	0.552 ± 0.012	0.775 ± 0.006	0.736 ± 0.008	0.814 ± 0.008	0.862 ± 0.011	0.179
SVM	0.544 ± 0.014	0.772 ± 0.007	0.742 ± 0.014	0.801 ± 0.009	0.870 ± 0.007	0.293
GB	0.544 ± 0.011	0.772 ± 0.005	0.743 ± 0.011	0.800 ± 0.020	0.862 ± 0.004	0.179
LR	0.536 ± 0.013	0.768 ± 0.006	0.753 ± 0.005	0.783 ± 0.010	0.863 ± 0.003	0.191
AB	0.524 ± 0.009	0.762 ± 0.005	0.747 ± 0.006	0.776 ± 0.008	0.793 ± 0.017	**0.000371**
KNN	0.508 ± 0.017	0.747 ± 0.008	0.625 ± 0.006	0.868 ± 0.012	0.826 ± 0.005	**0.010668**

Note: *p* < 0.05 shows a statistically significant difference between 4mCpred-EL and other method that is depicted in bold. Values are expressed as mean ± standard deviation.

## Supplementary Materials

The following are available online at https://www.mdpi.com/2073-4409/8/11/1332/s1.
