# Supplementary material for: 4mCpred-EL: An Ensemble Learning Framework for Identification of DNA N4-Methylcytosine Sites in the Mouse Genome
Source: Cells. 2019 Oct 28;8(11):1332. doi: 10.3390/cells8111332 (PMC6912380; doi:10.3390/cells8111332)
Supplement: Supplementary file 1 [file cells-08-01332-s001.pdf]

4mCpred-EL: an Ensemble Learning Framework for the Prediction of DNA N^4-methylcytosine Sites in the Mouse Genome

Balachandran Manavalan[1], *Shaherin Basith*[1], *Tae Hwan Shin*[1], *Da Yeon Lee*[1], *Leyi Wei*[2,*], *Gwang Lee*[1,*]

[1] Department of Physiology, Ajou University School of Medicine, Suwon, Republic of Korea, [2] School of Computer Science and Technology, Tianjin University, China

*** Corresponding authors**
E-mail: weileyi@tju.edu.cn and glee@ajou.ac.kr

Supporting Information

Supplementary tables

Table S1. The values of 15 DNA dinucleotide physicochemical properties

	Property	AA/TT	AC/GT	AG/CT	AT	CA/TG	CC/GG	CG	GA/TC	GC	TA
PC1	F-roll	0.04	0.06	0.04	0.05	0.04	0.04	0.04	0.05	0.05	0.03
PC2	F-tilt	0.08	0.07	0.06	0.1	0.06	0.06	0.06	0.07	0.07	0.07
PC3	F-twist	0.07	0.06	0.05	0.07	0.05	0.06	0.05	0.06	0.06	0.05
PC4	F-slide	6.69	6.8	3.47	9.61	2.00	2.99	2.71	4.27	4.21	1.85
PC5	F-shift	6.24	2.91	2.8	4.66	2.88	2.67	3.02	3.58	2.66	4.11
PC6	F-rise	21.34	21.98	17.48	24.79	14.51	14.25	14.66	18.41	17.31	14.24
PC7	Roll	1.05	2.01	3.6	0.61	5.60	4.68	6.02	2.44	1.70	3.50
PC8	Tilt	−1.26	0.33	−1.66	0	0.14	−0.77	0	1.44	0	0
PC9	Twist	35.02	31.53	32.29	30.72	35.43	33.54	33.67	35.67	34.07	36.94
PC10	Slide	−0.18	−0.59	−0.22	−0.68	0.48	−0.17	0.44	−0.05	−0.19	0.04
PC11	Shift	0.01	−0.02	−0.02	0	0.01	0.03	0	−0.01	0	0
PC12	Rise	3.25	3.24	3.32	3.21	3.37	3.36	3.29	3.30	3.27	3.39
PC13	Energy	−1.00	−1.44	−1.28	−0.88	−1.45	−1.84	−2.17	−1.30	−2.24	−0.58
PC14	Enthalpy	−7.60	−8.40	−7.80	−7.20	−8.50	−8.00	−10.60	−8.20	−9.80	−7.20
PC15	Entropy	−21.3	−22.4	−21.0	−20.4	−22.7	−19.9	−27.2	−22.20	−24.4	−21.3

Table S2. The values of 11 DNA trinucleotide physicochemical properties.

Trinucleotide	PC1	PC2	PC3	PC4	PC5	PC6	PC7	PC8	PC9	PC11	PC12
GGG	5.70	5.85	3	13	5.83	5.83	3.31	3.87	622.40	6.00	3.54
GGA	6.20	5.00	2	−5	4.99	4.99	3.82	3.58	622.40	3.80	4.80
GGC	8.20	9.10	3	45	9.08	9.08	1.39	2.45	622.40	10.00	1.31
GGT	5.20	5.30	2	8	5.32	5.32	3.62	4.16	622.40	5.40	3.88
GAG	6.60	6.00	2	8	5.98	5.98	3.22	3.35	621.40	5.40	3.88
GAA	5.10	4.05	1	−12	4.06	4.06	4.39	4.21	621.40	3.00	5.26
GAC	5.60	5.50	2	8	5.52	5.52	3.50	3.93	621.40	5.40	3.88
GAT	3.60	4.45	1	7	4.44	4.44	4.15	5.09	621.40	5.30	3.94
GCG	4.30	5.90	3	25	5.89	5.89	3.28	4.68	622.40	7.50	2.69
GCA	7.50	6.75	2	13	6.76	6.76	2.75	2.84	622.40	6.00	3.54
GCC	8.20	9.10	3	45	9.08	9.08	1.39	2.45	622.40	10.00	1.31
GCT	6.30	6.90	2	25	6.88	6.88	2.68	3.52	622.40	7.50	2.69
GTG	6.80	6.65	2	17	6.63	6.63	2.83	3.24	621.40	6.50	3.25
GTA	6.40	5.05	1	−6	5.07	5.07	3.77	3.47	621.40	3.70	4.86
GTC	5.60	5.50	2	8	5.52	5.52	3.50	3.93	621.40	5.40	3.88
GTT	1.60	2.65	1	−6	2.64	2.64	5.26	6.27	621.40	3.70	4.86
AGG	4.70	5.05	2	8	5.05	5.05	3.78	4.45	622.40	5.40	3.88
AGA	6.50	4.90	1	−9	4.89	4.89	3.88	3.41	622.40	3.30	5.09
AGC	6.30	6.90	2	25	6.88	6.88	2.68	3.52	622.40	7.50	2.69
AGT	2.00	3.90	1	11	3.92	3.92	4.47	6.03	622.40	5.80	3.65
AAG	4.20	4.70	1	6	4.70	4.70	4.00	4.74	621.40	5.20	3.99
AAA	0.10	0.05	0	−36	0.06	0.06	6.88	7.18	621.40	0.00	7.05
AAC	1.60	2.65	1	−6	2.64	2.64	5.26	6.27	621.40	3.70	4.86
AAT	0.00	0.35	0	−30	0.35	0.35	6.70	7.24	621.40	0.70	6.62
ACG	5.20	5.30	2	8	5.31	5.31	3.63	4.16	622.40	5.40	3.88
ACA	5.80	5.50	1	6	5.49	5.49	3.52	3.81	622.40	5.20	3.99
ACC	5.20	5.30	2	8	5.32	5.32	3.62	4.16	622.40	5.40	3.88
ACT	2.00	3.90	1	11	3.92	3.92	4.47	6.03	622.40	5.80	3.65
ATG	8.70	7.70	1	18	7.72	7.72	2.19	2.17	621.40	6.70	3.14
ATA	9.70	6.25	0	−13	6.27	6.27	3.05	1.61	621.40	2.80	5.38
ATC	3.60	4.45	1	7	4.44	4.44	4.15	5.09	621.40	5.30	3.94
ATT	0.00	0.35	0	−30	0.35	0.35	6.70	7.24	621.40	0.70	6.62
CGG	3.00	3.85	3	2	3.87	3.87	4.50	5.44	622.40	4.70	4.28
CGA	5.80	7.05	2	31	7.07	7.07	2.57	3.81	622.40	8.30	2.25
CGC	4.30	5.90	3	25	5.89	5.89	3.28	4.68	622.40	7.50	2.69
CGT	5.20	5.30	2	8	5.31	5.31	3.63	4.16	622.40	5.40	3.88
CAG	9.60	6.90	2	−2	6.90	6.90	2.67	1.67	621.40	4.20	4.57
CAA	6.20	4.75	1	−9	4.76	4.76	3.96	3.58	621.40	3.30	5.09
CAC	6.80	6.65	2	17	6.63	6.63	2.83	3.24	621.40	6.50	3.25

CAT	8.70	7.70	1	18	7.72	7.72	2.19	2.17	621.40	6.70	3.14
CCG	3.00	3.85	3	2	3.87	3.87	4.50	5.44	622.40	4.70	4.28
CCA	0.70	3.05	2	8	3.06	3.06	5.00	6.81	622.40	5.40	3.88
CCC	5.70	5.85	3	13	5.83	5.83	3.31	3.87	622.40	6.00	3.54
CCT	4.70	5.05	2	8	5.05	5.05	3.78	4.45	622.40	5.40	3.88
CTG	9.60	6.90	2	−2	6.90	6.90	2.67	1.67	621.40	4.20	4.57
CTA	7.80	5.00	1	−18	5.00	5.00	3.81	2.67	621.40	2.20	5.73
CTC	6.60	6.00	2	8	5.98	5.98	3.22	3.35	621.40	5.40	3.88
CTT	4.20	4.70	1	6	4.70	4.70	4.00	4.74	621.40	5.20	3.99
TGG	0.70	3.05	2	8	3.06	3.06	5.00	6.81	622.40	5.40	3.88
TGA	10.00	7.70	1	8	7.70	7.70	10.00	1.45	622.40	5.40	3.88
TGC	7.50	6.75	2	13	6.76	6.76	2.75	2.84	622.40	6.00	3.54
TGT	5.80	5.50	1	6	5.49	5.49	3.52	3.81	622.40	5.20	3.99
TAG	7.80	5.00	1	−18	5.00	5.00	3.81	2.67	621.40	2.20	5.73
TAA	7.30	4.65	0	−20	4.67	4.67	4.01	2.96	621.40	2.00	5.85
TAC	6.40	5.05	1	−6	5.07	5.07	3.77	3.47	621.40	3.70	4.86
TAT	9.70	6.25	0	−13	6.27	6.27	3.05	1.61	621.40	2.80	5.38
TCG	5.80	7.05	2	31	7.07	7.07	2.57	3.81	622.40	8.30	2.25
TCA	10.00	7.70	1	8	7.70	7.70	2.20	1.45	622.40	5.40	3.88
TCC	6.20	5.00	2	−5	4.99	4.99	3.82	3.58	622.40	3.80	4.80
TCT	6.50	4.90	1	−9	4.89	4.89	3.88	3.41	622.40	3.30	5.09
TTG	6.20	4.75	1	−9	4.76	4.76	3.96	3.58	621.40	3.30	5.09
TTA	7.30	4.65	0	−20	4.67	4.67	4.01	2.96	621.40	2.00	5.85
TTC	5.10	4.05	1	−12	4.06	4.06	4.39	4.21	621.40	3.00	5.26
TTT	0.10	0.05	0	−36	0.06	0.06	0.10	7.18	621.40	0.00	7.05

The first column represents the trinucleotides. PC1: Bendability (DNase); PC2: Bendability (consensus); PC3: Trinucleotide CG content; PC4: Nucleosome positioning; PC5: Consensus (roll), PC6: Consensus (rigid), PC7: DNase I; PC8: DNase I (rigid); PC9: Molecular weight (Daltons); PC10: Nucleosome; PC11: Nucleosome (rigid).

Table S3. The performance of different classifiers on seven different feature encodings using benchmarking dataset

Classifier	Encoding	MCC	ACC	SN	SP	AUC
RF	BPF	0.369±0.025	0.684±0.018	0.678±0.031	0.690±0.025	0.731±0.024
	DPCP	0.508±0.010	0.754±0.005	0.736±0.009	0.772±0.007	0.816±0.004
	EIIP	0.556±0.029	0.778±0.015	0.760±0.022	0.796±0.018	0.851±0.028
	Kmer	0.558±0.009	0.779±0.004	0.762±0.014	0.796±0.008	0.849±0.007
	M6AMRFS	0.385±0.016	0.692±0.008	0.700±0.011	0.685±0.008	0.744±0.010
	RFHC	0.384±0.015	0.692±0.008	0.710±0.007	0.674±0.011	0.738±0.009
	TPCP	0.539±0.010	0.769±0.005	0.750±0.005	0.788±0.008	0.836±0.006
SVM	BPF	0.393±0.015	0.696±0.007	0.692±0.028	0.701±0.027	0.715±0.077
	DPCP	0.547±0.003	0.773±0.001	0.757±0.004	0.789±0.002	0.845±0.005
	EIIP	0.545±0.017	0.772±0.009	0.761±0.012	0.784±0.007	0.841±0.006
	Kmer	0.559±0.005	0.780±0.002	0.775±0.004	0.784±0.005	0.854±0.003
	M6AMRFS	0.394±0.012	0.697±0.006	0.704±0.011	0.689±0.008	0.757±0.011
	RFHC	0.403±0.014	0.701±0.007	0.693±0.030	0.709±0.030	0.714±0.090
	TPCP	0.557±0.006	0.778±0.003	0.766±0.003	0.790±0.007	0.846±0.004
ERT	BPF	0.415±0.010	0.708±0.005	0.708±0.012	0.707±0.008	0.757±0.011
	DPCP	0.517±0.009	0.758±0.005	0.731±0.010	0.786±0.009	0.822±0.004
	EIIP	0.541±0.005	0.771±0.002	0.751±0.004	0.790±0.004	0.836±0.004
	Kmer	0.559±0.008	0.779±0.004	0.758±0.008	0.800±0.004	0.848±0.005
	M6AMRFS	0.393±0.011	0.696±0.006	0.700±0.007	0.692±0.012	0.748±0.008
	RFHC	0.409±0.008	0.704±0.004	0.708±0.004	0.701±0.010	0.755±0.010
	TPCP	0.415±0.010	0.708±0.005	0.708±0.012	0.707±0.008	0.757±0.011
GB	BPF	0.395±0.014	0.698±0.007	0.706±0.009	0.689±0.012	0.751±0.009
	DPCP	0.502±0.013	0.751±0.006	0.733±0.006	0.768±0.010	0.809±0.006
	EIIP	0.526±0.006	0.763±0.003	0.747±0.006	0.779±0.003	0.833±0.004
	Kmer	0.556±0.011	0.778±0.006	0.762±0.011	0.793±0.002	0.845±0.010
	M6AMRFS	0.372±0.013	0.686±0.006	0.693±0.011	0.679±0.006	0.741±0.006
	RFHC	0.375±0.016	0.688±0.008	0.699±0.014	0.676±0.010	0.741±0.011
	TPCP	0.533±0.014	0.766±0.007	0.754±0.011	0.779±0.011	0.836±0.009
AB	BPF	0.326±0.009	0.663±0.005	0.679±0.010	0.646±0.011	0.663±0.005
	DPCP	0.499±0.009	0.750±0.004	0.735±0.008	0.764±0.006	0.750±0.004
	EIIP	0.514±0.012	0.757±0.006	0.746±0.005	0.768±0.011	0.760±0.008
	Kmer	0.537±0.014	0.769±0.007	0.755±0.010	0.782±0.006	0.777±0.008
	M6AMRFS	0.344±0.011	0.672±0.005	0.702±0.008	0.641±0.002	0.673±0.004
	RFHC	0.342±0.010	0.671±0.005	0.692±0.006	0.649±0.006	0.672±0.006
	TPCP	0.523±0.010	0.762±0.005	0.749±0.006	0.774±0.008	0.766±0.005
KNN	BPF	0.347±0.012	0.673±0.006	0.707±0.014	0.639±0.024	0.733±0.005
	DPCP	0.474±0.003	0.736±0.002	0.692±0.010	0.780±0.006	0.815±0.003
	EIIP	0.469±0.014	0.726±0.007	0.593±0.011	0.859±0.004	0.820±0.002
	Kmer	0.472±0.020	0.735±0.010	0.699±0.016	0.772±0.008	0.812±0.003
	M6AMRFS	0.355±0.017	0.677±0.008	0.710±0.034	0.644±0.026	0.740±0.008
	RFHC	0.355±0.014	0.677±0.007	0.711±0.016	0.642±0.027	0.738±0.008
	TPCP	0.488±0.011	0.738±0.006	0.621±0.016	0.854±0.004	0.828±0.001
LR	BPF	0.301±0.013	0.650±0.006	0.662±0.006	0.639±0.013	0.701±0.005
	DPCP	0.518±0.004	0.759±0.002	0.756±0.006	0.762±0.006	0.835±0.003
	EIIP	0.523±0.019	0.762±0.010	0.762±0.011	0.761±0.008	0.836±0.007

	Kmer	0.535±0.005	0.767±0.002	0.759±0.005	0.775±0.002	0.844±0.004
	M6AMRFS	0.313±0.010	0.657±0.005	0.669±0.005	0.644±0.006	0.715±0.008
	RFHC	0.307±0.012	0.653±0.006	0.669±0.007	0.638±0.012	0.715±0.004
	TPCP	0.537±0.003	0.768±0.002	0.758±0.003	0.778±0.001	0.842±0.004
